# Health-related quality of life in a clinical sample of obese children and adolescents

**DOI:** 10.1186/1477-7525-8-134

**Published:** 2010-11-15

**Authors:** Afsane Riazi, Sania Shakoor, Isobel Dundas, Christine Eiser, Sheila A McKenzie

**Affiliations:** 1 Department of Psychology, Royal Holloway, University of London, Surrey, UK; 2 Social, Genetic, and Developmental Psychiatry, Institute of Psychiatry, London, UK; 3 Department of Pediatric Respiratory Medicine, Royal London Hospital, London, UK; 4 Department of Psychology, University of Sheffield, Sheffield, UK

## Abstract

**Background:**

Obesity affects ethnic minority groups disproportionately, especially in the pediatric population. However, little is known about the impact of obesity on health-related quality of life (HRQoL) in children and adolescents from mixed-ethnic samples. The purpose of this study was to: 1) measure HRQoL in a mixed-ethnic clinical sample of obese children and adolescents, 2) compare HRQoL assessments in obese participants and healthy controls, and 3) compare HRQoL in obese children and adolescents according to their pubertal status.

**Methods:**

A clinical sample of children and adolescents with obesity (n = 96) and healthy children and adolescents attending local schools (n = 444) completed the Pediatric Quality of Life Inventory (PedsQL; UK version 4). Age-appropriate versions were self-administered by children and adolescents aged 8-18 years, and interview administered to children aged 5-7 years. Multiple regression analyses controlling for age, gender, pubertal status, and ethnicity were used to compare the PedsQL scores of the two samples.

**Results:**

The clinical sample of obese children and adolescents had poorer HRQoL scores on all dimensions of the PedsQL compared to the healthy controls (p < 0.005). Subsequent analyses also demonstrated that in this sample of mixed-ethnic children and adolescents, prepubescent obese children achieved the poorest scores in the emotional functioning dimension.

**Conclusions:**

Obesity significantly impacts on physical, emotional, social and school functioning of mixed-ethnic children and adolescents. Clinicians need to be aware of the significant impact of obesity on all aspects of functioning. More effort is required to target interventions to improve the quality of life of children with obesity.

## Background

Obesity in children and adolescents adversely affects both their psychological as well as their physical health. When compared to non-obese children, obese children feel they are less competent in their social and athletic abilities as well as less attractive and worthwhile [1]. These feelings may be aggravated by discrimination and teasing by peers [2].

Health-related quality of life (HRQoL) is a comprehensive and multi-dimensional construct that includes physical, emotional, and social functioning. For children and adolescents, cognitive functioning is often also included [3]. Recently the impact of obesity on HRQoL in children and adolescents has been demonstrated in both community-based [4,5] and clinical samples [6,7]. In both children and adolescents, obesity seems to affect physical functioning most strongly, but some studies have shown that emotional and social functioning are also significantly affected [4,6], with adolescent-reported emotional functioning being most impaired in the 12-14 age group [8]. A recent comprehensive review suggests that increasing weight status has a moderate to strong negative influence on overall HRQoL in paediatric populations, with decrements in HRQoL being evident as soon as BMI is above the normal range [9]. The same review found an inverse linear relationship between HRQoL and BMI for most studies [9].

There has been a disproportionate increase in obesity in non-white compared to white children [10]. For example, in east London, UK, where just under 40% of the population are non-white [11], around 20% of adolescent boys and 22% of adolescent girls are obese [12], and Asian children are four times more likely to be obese than those who are white [13]. These differences may be attributed to genetics and inter-generational gene-environmental interactions, as well as different patterns and cultural norms that do not recognise obesity as a problem [14]. Successful interventions to reduce obesity needs to take into account the social and cultural context in which obesity occurs, and thus the importance of studying obese children from non-white backgrounds cannot be undermined. Yet little is known about the effect of obesity on non-white children and adolescents with obesity, especially in the UK. One study by Hughes [7] examined HRQoL in a pediatric obese clinical sample in the UK, but the adverse impact of obesity on HRQoL in non-white children was not apparent. Since there is also evidence that children and adolescents from some ethnic groups (eg Bangladeshi in the UK), have lower rates of psychological distress, despite their higher levels of social disadvantage [15,16], there is a need to identify whether obesity has a significant impact on physical and psychological functioning in a mixed-ethnic clinical sample of obese children and adolescents. Additionally, as there are reports of increasing levels of obesity in very young children [17], the effect of obesity needs to be examined in a wide age range that includes children younger than some previous reports [7].

Further to social and cultural factors in which obesity occurs, pubertal status may also influence the associations between obesity and HRQoL. The relationship between puberty and body weight is reported to be interrelated [18], whereby pubertal changes (i.e. increases in sex hormones) can contribute towards increased body weight and increases in body weight can contribute towards the onset of puberty [19]. Furthermore, pubertal status has also been shown to have an impact on psychosocial functioning, especially in girls [20], thus identifying puberty as an influential factor affecting both body weight and HRQoL. We examined pubertal status and its impact on obesity and HRQoL.

The aims of this study were therefore to: 1) to measure HRQoL in a mixed-ethnic clinical sample of obese children and adolescents and to observe any differences in the impact of obesity on HRQoL according to different ethnic groups as well as gender, 2) to compare HRQoL assessments in obese participants and healthy controls taking into account their demographic status and 3) compare HRQoL in obese children and adolescents according to their pubertal status. Based on previous literature, we specifically hypothesised that: 1) obese children and adolescents will report worse HRQoL scores than healthy control group matched for gender, ethnicity and age, 2) obese prepubescent children will report better HRQoL compared to obese children and adolescents in puberty or in the postpubertal stage, 3) within the obese sample, higher BMI scores will be associated with more decrements in HRQoL scores.

## Methods

### Participants

Obese children and adolescents aged between 5 and 16 years were invited to participate. These were consecutive attenders at the Paediatric Obesity Service, Royal London Hospital, for the evaluation of medical complications of obesity. Children were excluded if they had any genetic syndromes associated with obesity, including cerebral palsy, spina bifida, and hypothyroidism.

Controls were healthy children and adolescents aged 5 to 16 years recruited from 12 local schools (8 primary and 4 secondary schools) in the east London district of Tower Hamlets. Parents were sent an information sheet about the study and a reply slip with a consent form to let their children take part in the study. Height and weight were measured from all participants, who also completed the HRQoL measure [21], either in the clinic or in the school setting.

### Health-related quality of life

A UK-version of a generic pediatric QOL inventory (PedsQL 4.0) [21] was used to measure HRQoL. This scale includes 23 items organised around four domains (physical functioning, emotional functioning, social functioning, and school functioning). Three versions of the scale were used: for young children aged 5 to 7 years, the measure was administered by an interviewer [SS] and had a three-point response scale (0 = not at all a problem, 2 = sometimes and 4 = a lot), with each response choice anchored to either a smiling, middle or frowning face; for children aged 8 to 12 years, and teenagers aged 13 to 18 years, the self-report scale had a five-point response scale (0 = never a problem, 1 = almost never, 2 = sometimes, 3 = often and 4 = almost always). Items are linearly transformed to a 0-100 scale, so that higher scores indicate better HRQoL. The same researcher [SS] was present at both the clinic and school settings.

### Pubertal status self-report

The adapted version of the Self-rating Scale for Pubertal Development [22,23] was used to assess pubertal status. The scale uses body hair growth, voice changes and facial hair growth for boys, and body hair growth, breast development and menarche for girls, to categorise respondents into the following pubertal categories: prepubertal, early pubertal, midpubertal, late pubertal, and postpubertal. For the purpose of the statistical analyses for the present study, all categories from early pubertal to postpubertal status were combined to form one group (pubertal group) and compared with the prepubertal group.

### Anthropometry

Height was measured to the nearest 1 mm using a wall-mounted portable stadiometer (SECA). Weight was measured whilst dressed to the nearest 0.1 kg using scale (EKS). BMI was calculated as weight (kg)/height (m^2^) and converted to z scores for age using the Child Growth Foundation data [24].

Written parental informed consent and child assent were obtained before participation in the study. The project was approved by the East London and the City Research Ethics Committee.

### Statistical analyses

Independent t-tests and chi-square tests were used to compare demographic variables in the two groups. Due to differences in both age and ethnicity between the two samples, a matched control analysis was first conducted. The two samples were matched for gender, ethnicity and age, and paired sample t-tests were used to examine differences between the samples. This was done by randomly selecting participants from the control sample who matched the clinic sample for these three variables. Next, multiple regression analyses controlling for age, gender, pubertal status and ethnicity were used to compare the PedsQL scores of the clinic and the control samples. Finally, analysis of covariance was used to investigate the interaction effect of pubertal status and obesity on PedsQL scores, as well as the interaction effects of ethnicity and obesity, and gender and obesity on PedsQL scores.

## Results

### Sample characteristics (Table 1)

Over the study period, 112 children attended clinic and were eligible to take part. Sixteen children and adolescents who fitted the inclusion criteria did not attend the clinic. A total of 96 consecutive attenders took part. There were no refusals. Data were collected from 448 pupils from local school

The ethnic distribution of the obese clinical sample was similar to other paediatric obese distribution reported in east London. The proportion of participants from white and Afro-Caribbean backgrounds was smaller in the control group. The obese clinical group were also slightly older than the control group. No significant differences in demographic variables were found between clinic attenders and non-attenders (Table 1).

**Table 1:** Demographic variables

	Obese group (n = 96)	Control group (n = 444)	p-value
Age	11.5 (2.9)	10.3 (2.6)	0.000
Gender			
Female	50 (52.1%)	251 (56.5%)	0.247
Male	46 (47.9%)	193 (43.5%)	
Ethnicity			
White	28 (28.1%)	81 (18.2%)	0.000
Black	13 (13.5%)	28 (6.3%)	
Asian	46 (47.5%)	319 (70.9%)	
Other	10 (10.4%)	20 (4.5%)	
Weight (kg)	83.1 (31.4)	36.6 (12.3)	0.000
BMIz	3.5 (0.5)	0.3 (1.4)	0.000
			

Data are mean (s.d) or frequency

### Paired matched comparisons of HRQoL in the obese vs control samples

The results of the matched control analysis (n = 83) demonstrated that children and adolescents with obesity reported significantly lower HRQoL scores on all dimensions of the PedsQL (physical functioning, emotional functioning, social functioning, school functioning, psychosocial health, and total scale score) compared to the matched control sample (p < 0.005) (Table 2). This suggests that obesity has a significant impact on children and adolescents compared to a comparative group matched for gender, age and ethnicity.

**Table 2:** Matched pairs comparisons of PedsQL scores for the obese clinical group and the healthy control group

	Obese clinical sample (n = 83)	Control sample (n = 83)	*t (df)*	Paired samples t-tests p-value
Physical functioning	70.1 (17.0)	82.8 (12.4)	-5.5 (82)	< 0.001
Emotional functioning	61.4 (20.8)	72.8 (17.8)	-3.9 (82)	< 0.001
Social functioning	72.8 (20.1)	81.7 (16.4)	-3.4 (82)	0.001
School functioning	65.4 (19.9)	73.1 (16.8)	-3.0 (82)	0.004
Psychosocial health	66.6 (16.3)	75.9 (12.7)	-4.3 (82)	< 0.001
Total scale score	67.4 (15.3)	78.3 (11.3)	-5.4 (82)	< 0.001

Mean (sd)

### Comparison of HRQoL scores in the obese vs control samples controlling for demographic variables

A similar result was obtained using the multiple regression analyses. Controlling for age, gender, pubertal status and ethnicity, the obese clinical group reported lower HRQoL scores on all dimensions of the PedsQL compared to the control group (*p *< 0.005) (Table 3). Pubertal status also had an effect on several PedsQL dimensions (social functioning, psychosocial health and total score), with prepubescent children of both groups reporting poorer functioning on these dimensions (Table 4). An interaction effect of group and pubertal status was seen on the emotional functioning dimension only, with the prepubescent obese children achieving particularly poorer scores in this dimension (Table 4). Interaction effects of group and gender, and group and ethnicity were not found (data not shown).

**Table 3:** PedsQL scores for obese clinical sample compared with control sample controlling for gender, age, pubertal status and ethnicity

	Obese clinical sample (n = 96)	Control sample (n = 444)	*b*	*SE b*	Multiple Regression p-value
Physical functioning	68.9 (65.7 - 72.1)	80.1 (78.7 - 81.6)	11.2	1.79	< 0.001
Emotional functioning	61.5 (57.6 - 65.4)	73.0 (71.2 - 74.8)	11.5	2.22	< 0.001
Social functioning	69.8 (66.1 - 73.6)	79.5 (77.8 - 81.2)	9.69	2.13	< 0.001
School functioning	64.4 (60.6 - 68.2)	70.9 (69.2 - 72.7)	6.54	2.2	0.003
Psychosocial health	65.3 (62.2 - 68.3)	74.5 (73.1 - 75.9)	9.2	1.7	< 0.001
Total scale score	66.2 (63.4 - 69.0)	76.5 (75.2 - 77.7)	10.3	1.6	< 0.001

Mean (95%CI)

**Table 4:** PedsQL scores according to sample and pubertal status controlling for gender, age and ethnicity

	Obese group (n = 96)		Control group (n = 444)		Analysis of Covariance Group effect	Analysis of Covariance Pubertal status effect	Analysis of Covariance p-value group × pubertal status interaction
	**Prepubescent (n = 40)**	**Early to post pubertal (n = 56)**	**Prepubescent (n = 312)**	**Early to post pubertal (n = 132)**	***F; p-value***	***F; p-value***	***F; p-value***

Physical functioning	68.7 (63.8 -73.6)	70.9 (66.5 - 75.2)	78.6 (76.7 - 80.6)	82.9 (79.7 - 86.2)	37.9; < 0.001	2.0; ns	0.3; ns
Emotional functioning	56.5 (50.5 - 62.6)	66.5 (61.1 - 71.8)	72.7 (70.3 - 75.1)	73.2 (69.2 - 77.2)	26.8; < 0.001	3.4; < 0.06	4.6; < 0.05
Social functioning	67.1 (61.3 - 72.8)	77.3 (72.1 - 82.5)	74.6 (72.3 - 76.9)	88.9 (85.0 - 92.7)	20.3; < 0.001	20.7 < 0.001	0.9; ns
School functioning	65.0 (59.1 - 70.9)	65.5 (60.2 - 70.8)	69.4 (67.1 - 71.8)	73.9 (70.0 - 77.8)	8.7; 0.003	0.8; ns	0.8; ns
Psychosocial health	62.9 (58.1 - 67.6)	69.8 (65.6 - 74.0)	72.2 (70.3 - 74.1)	78.7 (75.5 - 81.8)	27.6; < 0.001	9.2; 0.003	0.0; ns
Total scale score	64.8 (60.5- 69.2)	69.7 (65.8 - 73.5)	74.4 (72.6 - 76.1)	80.3 (77.4 - 83.2)	40.3; < 0.001	7.0; 0.008	0.1; ns

### The relationship between BMIz and HRQoL scores

We also examined the relationship between BMIz scores and each of the PedsQL subscales controlling for age, gender, pubertal status and ethnicity in the total sample and in the obese clinical group separately. In the total sample, BMIz score was significantly associated with all PedsQL subscales (p < 0.05) except school functioning. In the obese group, BMIz scores were not significantly associated with any of the PedsQL subscales. Quadratic terms were added to the equations but these did not prove to be significant for all PedsQL subscales, except physical functioning (p < 0.05). However, although PedsQL scores in the obese clinical group demonstrated sufficient variability in scores, the range of BMIz scores in this group was much narrower (data not shown).

## Discussion

In the present study using self-report measures, obese children and adolescents were significantly compromised in all HRQoL dimensions compared to non-obese controls. The findings are consistent with another study using a clinical sample [6] that also found significant impairment in all HRQoL dimensions in the obese participants (5-16 years) compared to non-obese controls. However the results are in contrast to another study using a clinical sample that found only physical health to be significantly impaired in obese children aged 8-12 years [7]. A recent comprehensive review on HRQoL in obese children and adolescents also suggests that although physical and social functioning are mostly affected, there is some evidence of decrements in emotional functioning, and minimal evidence of impaired school functioning [9]. Our study thus supports a minority of studies using clinical samples that demonstrate impaired school functioning in obese children and adolescents, perhaps suggesting that individuals seeking treatment may experience more impairment [9].

In our present study, the pre-pubescent obese children reported the poorest emotional functioning. This finding is novel and requires further investigation, as it has been suggested that it is in fact, early adolescence that may be a particularly vulnerable period of HRQoL impairments in obese youngsters [9]. Although adults with obesity do not show marked decrease in emotional functioning compared to healthy controls [25], the findings here suggest that that the impact of obesity on emotional health in prepubescent children cannot be overlooked. This is an interesting finding, considering that our sample consisted of a large proportion of Bangladeshi children in east London, who have been found to have good mental health despite social deprivation [15,16]. High levels of family support and high ethnic density have been suggested as possible protective factors on mental health in this sample [16]. Thus it may be that the effect of obesity could override any ethnically related protective factors in young children, although our findings require further investigations.

It has been suggested that the psychosocial aspects of obesity, which are often ignored in the drive to improve physical health, are particularly important in children, and that the first problems caused by obesity in childhood are likely to be emotional and psychological [26]. It is not clear from our study whether the effects on mental health are influenced by social factors, such as teasing or bullying by peers, since there were no combined effects of obesity and pubertal status on social functioning. Whatever the reason, coupled with the increasing prevalence of obesity, we suggest that parents, clinicians, teachers, and others who come into contact with children, are aware of the wide ranging impact of obesity. Our results also demonstrated that the degree of obesity was not related to the degree of psychosocial functioning. This implies that once an individual is obese it does not matter how obese they are, they are likely to have reduced psychosocial functioning. This has clear implications for designing effective interventions, as it needs to be targeted to all obese children, and not just those who are severely obese. This is in contrast to our hypothesis that higher BMI scores will be associated with more decrements in HRQoL scores. However, this lack of association may be due to the lack of variability in BMIz scores in our obese sample, as has been found in some previous studies with a narrow range of BMI scores [9].

We also analysed the impact of obesity on HRQoL by gender, but found the results to be similar for boys and girls. This is in line with previous studies [5-7] and suggests that the impact of obesity is not necessarily gender-specific. Nor did the effect of obesity on HRQoL differ by ethnicity. However, the subsample analyses may have been affected by the relatively small sample size of the obese group.

Several limitations of the study should be noted. First, parent-proxy report scores were not collected. However, it has been suggested that even very young children are able to provide self-report data, and that self-report data are preferable as they provide a more accurate picture of children's quality of life [27]. Second, pubertal status was also collected through self-report, and this was not supplemented by physical examination. Although the correlations between self-reported pubertal status and physician examination are normally in the moderate to high range, there is some evidence that self-assessment of pubertal stage in overweight children may not be as reliable [28]. Third, the present study included a clinical sample of obese children and adolescents who were referred for investigations into complications of obesity. Therefore, the results of the present study may not be applicable to children and adolescents in the community. Fourth, although obese youngsters from mixed ethnic background demonstrate significantly impaired HRQoL it is nevertheless difficult to interpret the findings in light of the group's ethnic makeup itself.

In conclusion, the present study demonstrated that a mixed-ethnic clinical sample of children and adolescents with obesity report significantly lower HRQoL scores compared to a control group of children and adolescents. The emotional impact of obesity in prepubescent children cannot be underestimated, although this finding requires further investigations. Finally, this study employed a generic version of HRQoL measure. Although there are advantages to using generic measures, such as the ability to compare scores to the normative sample [29], a more condition-specific measure may capture the impact of obesity in children and adolescents more accurately, and be more responsive to any intervention-related changes in HRQoL [30].

## Conclusions

This is one of the first studies to examine health-related quality of life in children and adolescents in a mixed-ethnic sample in the UK. This study demonstrated that obese children and adolescents were significantly compromised in all HRQoL dimensions compared to non-obese controls. The study also examined the effect of obesity in a wide age range that includes children younger than some previous reports, and demonstrated that pre-pubescent obese children report the poorest emotional functioning.

## Competing interests

The authors declare that they have no competing interests.

## Authors' contributions

AR conceived and designed the study, analysed and interpreted the data, and drafted the manuscript. SS and ID collected the data. ID, CE and SM were involved in guiding the study including the design and coordination. All authors contributed to the interpretation of data and writing of the manuscript. All authors read and approved the final manuscript.
